# Induced Pluripotent Stem Cells Inhibit Bleomycin-Induced Pulmonary Fibrosis in Mice through Suppressing TGF-β1/Smad-Mediated Epithelial to Mesenchymal Transition

**DOI:** 10.3389/fphar.2016.00430

**Published:** 2016-11-15

**Authors:** Yan Zhou, Zhong He, Yuan Gao, Rui Zheng, Xiaoye Zhang, Li Zhao, Mingqi Tan

**Affiliations:** ^1^Department of Respiratory Medicine, Shengjing Hospital of China Medical UniversityShenyang, China; ^2^Department of Oncology, Shengjing Hospital of China Medical UniversityShenyang, China

**Keywords:** bleomycin, epithelial to mesenchymal transition (EMT), induced pluripotent stem (iPS) cells, inflammation, pulmonary fibrosis, TGF-β1

## Abstract

Pulmonary fibrosis is a progressive and irreversible fibrotic lung disorder with high mortality and few treatment options. Recently, induced pluripotent stem (iPS) cells have been considered as an ideal resource for stem cell-based therapy. Although, an earlier study demonstrated the therapeutic effect of iPS cells on pulmonary fibrosis, the exact mechanisms remain obscure. The present study investigated the effects of iPS cells on inflammatory responses, transforming growth factor (TGF)-β1 signaling pathway, and epithelial to mesenchymal transition (EMT) during bleomycin (BLM)-induced lung fibrosis. A single intratracheal instillation of BLM (5 mg/kg) was performed to induce pulmonary fibrosis in C57BL/6 mice. Then, iPS cells (c-Myc-free) were administrated intravenously at 24 h following BLM instillation. Three weeks after BLM administration, pulmonary fibrosis was evaluated. As expected, treatment with iPS cells significantly limited the pathological changes, edema, and collagen deposition in lung tissues of BLM-induced mice. Mechanically, treatment with iPS cells obviously repressed the expression ratios of matrix metalloproteinase-2 (MMP-2) to its tissue inhibitor -2 (TIMP-2) and MMP-9/TIMP-1 in BLM-induced pulmonary tissues. In addition, iPS cell administration remarkably suppressed BLM-induced up-regulation of pulmonary inflammatory mediators, including tumor necrosis factor-α, interleukin (IL)-1β, IL-6, inducible nitric oxide synthase, nitric oxide, cyclooxygenase-2 and prostaglandin E_2_. We further demonstrated that transplantation of iPS cells markedly inhibited BLM-mediated activation of TGF-β1/Mothers against decapentaplegic homolog 2/3 (Smad2/3) and EMT in lung tissues through up-regulating epithelial marker E-cadherin and down-regulating mesenchymal markers including fibronectin, vimentin and α-smooth muscle actin. Moreover, *in vitro*, iPS cell-conditioned medium (iPSC-CM) profoundly inhibited TGF-β1-induced EMT signaling pathway in mouse alveolar epithelial type II cells (AECII). Collectively, our results suggest that transplantation of iPS cells could suppress inflammatory responses, TGF-β1/Smad2/3 pathway and EMT during the progression of BLM-induced pulmonary fibrosis, providing new useful clues regarding the mechanisms of iPS cells in the treatment for this disease.

## Introduction

Pulmonary fibrosis is a chronic interstitial lung disease, which is characterized by inflammatory injury of alveolar epithelial cells, excessive proliferation of fibroblasts, aberrant deposition of extracellular matrix (EMC), and abnormal repair and remodeling of lung tissue (White et al., 2003). These pathologic changes result in progressive decline in pulmonary function, eventually leading to respiratory failure (Wynn, 2011). Previous studies have suggested that pulmonary inflammation is an early event during the development of pulmonary fibrosis, as evidence by the release of various inflammatory mediators (Kandhare et al., 2015). Anti-inflammation agents like cytokines antagonists, nuclear factor (NF)-κB antagonists and inducible nitric oxide synthase (iNOS) inhibitors have been proposed as treatment option for lung fibrosis (Woodcock and Maher, 2014). Emerging evidence indicates that epithelial to mesenchymal transition (EMT) has a critical role in the progression of fibrotic lung diseases due to its contribution to the generation of numerous ECM-producing fibroblasts/myofibroblasts (Andersson-Sjoland et al., 2008; Omenetti et al., 2008). Transforming growth factor (TGF)-β1/Smad2/3 signaling is one of the main pathways involved in a variety of pulmonary fibrogenesis processes, including inflammation, EMT, and ECM deposition. In the past few years, TGF-β1 pathway has attracted considerable attention from researchers as a therapeutic target for lung fibrosis (D’Alessandro-Gabazza et al., 2012; Dong et al., 2012). However, although various potential therapeutics for pulmonary fibrosis have been widely applied in clinical trials, this disease still has a high mortality rate of up to 40% within 5 years of diagnosis (Antoniou et al., 2013; Gomer, 2013). Therefore, exploring new effective strategies for ameliorating pulmonary fibrosis is a top priority.

Stem cell-based approaches, including the use of mesenchymal stem cells (MSCs) or embryonic stem (ES) cells, have been widely studied for their effects in treating pulmonary fibrosis (Ortiz et al., 2003; Moodley et al., 2009; Banerjee et al., 2012; Spitalieri et al., 2012). However, there are some concerns with regards to the efficiency and availability of MSCs due to their limited life span when cultured *in vitro* (Pozzobon et al., 2010). Additionally, the problems of ethical concerns and immune rejection following transplant restrict the clinical application of ES cells. Recently, induced pluripotent stem (iPS) cells, derived from adult somatic cells via reprogramming by introduction of specific pluripotency genes, have opened a novel avenue for stem cell-based therapies (Takahashi et al., 2007). iPS cells share the similar characteristics such as morphology, self-renewal abilities, gene expression, and surface antigens with ES cells and avoid the ethical and immunorejective problems. More importantly, a recent report has found that iPS cells have more potent immunomodulatory effects compared with bone marrow-derived MSCs *in vitro* (Schnabel et al., 2014). Accordingly, iPS cells could be suggested as quite promising candidates for cell therapies and regenerative medicine.

In general, introduction of four genes, including Oct4, Sox2, Klf4 and c-Myc, to somatic cells results in generation of iPS cells, which can lead to pluripotency comparable with ES cells. Surprisingly, recent studies have successfully generated 3-gene iPS cells (c-Myc-free) from fibroblasts, which were shown low tumorigenic risk (Li et al., 2011; Yang et al., 2011). Notably, How et al. (2013) demonstrated that 3-gene iPS cell transplantation could attenuate bleomycin (BLM)-induced pulmonary fibrosis partially via the early amelioration of inflammation. However, whether other mechanisms are involved in the therapeutic effects of specific-iPS cells on lung fibrosis remains uncertain. In the present study, we hypothesized that iPS cell transplantation could mitigate TGF-β1/Smad2/3 signaling pathway as well as EMT process during pulmonary fibrosis. To test the hypothesis, we developed a BLM-induced pulmonary fibrosis mouse model, and demonstrated that not only amelioration of pulmonary inflammation but also inhibition of TGF-β1 signaling and EMT process were involved in the anti-fibrotic effects of iPS cells.

## Materials and Methods

### Animals

C57BL/6 mice were purchased from Vital River Laboratory Animal Technology, Co. Ltd (Beijing, China). The mice were raised under controlled conditions (temperature 22 ± 1°C and humidity 40–60%) with 12 h dark/light cycle. All animal care and experimental protocols were approved by the Animal Care Ethics and Use Committee of China Medical University and performed in accordance with the guidelines of this committee.

### The Isolation of Mouse Embryonic Fibroblasts (MEFs)

Mouse embryonic fibroblasts were isolated from 12.5∼14.5-day-old embryos of female C57BL/6 mice. Briefly, embryos were separated from the uterus of mice at day 12.5∼14.5 post-conception, and the viscera and head of each embryo were removed. Then, the remaining embryos were washed with phosphate-buffered saline (PBS), minced into small pieces (1–3 mm^3^), digested with 0.25% trypsin-EDTA (Sigma-Aldrich, St. Louis, MO, USA), and incubated in Dulbecco’s Modified Eagle’s Medium (DMEM; Gibco, MD, USA) supplemented with 10% fetal bovine serum (FBS; Hyclone, USA) and 1% penicillin-streptomycin at 37°C in humidified atmosphere with 5% CO_2_. After filtering and centrifuging, the cells were counted and seeded in 12-well plates for the following experiments.

### The Generation of Mouse iPS Cells and the Culture of ES Cells

Mouse iPS cells were generated from MEFs and reprogrammed according to previous studies with minor modification (How et al., 2013). Briefly, MEFs were seeded into 6-well plates and cultured overnight in fibroblasts medium consisting of DMEM, 10% FBS and 1% penicillin-streptomycin. When reached 70% confluence, cells were transfected with lentiviral vectors (Hanbio, Shanghai, China) encoding three transcription factors including Oct4, Sox2, and Klf4 in the presence of ploybrene (4 μg/ml; Sigma-Aldrich, St. Louis, MO, USA). After 24 h of infection, the cells were released in fresh medium. Forty-eight hours after transduction, the cells were cultured using mouse iPS cell medium which consists of DMEM, 15% FBS, 2 mM glutamine, 0.1 mM non-essential amino acids (NEAAs), and 0.1 mM β-mercaptoethanol, and the medium was changed every other day. Subsequently, the iPS cells formed clones on MEFs and the clones were subjected to expansion for other experiments.

Mouse ES cells were purchased from Shanghai Institute of Cell Resource Center of Life Science (Shanghai, China). Cells were cultured in DMEM medium supplemented with 15% FBS (Invitrogen, Carlsbad, CA, USA), 1% NEAA, 0.25% β-mercaptoethanol, and 1% LIF (1000 U/ml; Millipore, MA, USA) at 37°C in humidified atmosphere with 5% CO_2_.

### The Preparation of the iPS Cell-Conditioned Medium (iPSC-CM)

The well-grown iPS cells were seeded into 6-well plates for 24 h, and then cultured with FBS-free DMEM supplemented with 2 mM glutamine, 0.1 mM NEAAs, and 0.1 mM β-mercaptoethanol. After 48 h of incubation, the culture medium were collected and centrifuged at 1500 rpm for 10 min. The supernatant was obtained to be used as the iPSC-CM in subsequent *in vitro* experiments.

### The Isolation and Treatment of Mouse Alveolar Epithelial Type II Cells (AECII)

Primary mouse AECII isolation was conducted as previously described (Mutze et al., 2015). In brief, the lungs of female C57BL/6 mice were washed with PBS and inflated with 0.25% trypsin followed by incubation for 20 min at 37°C. Subsequently, the lungs were minced into small pieces, filtered through 100 and 60 μm stainless meshes, and the cell suspension was centrifuged at 1000 rpm at 4°C for 10 min. After re-suspension in DMEM, the cells underwent percoll gradient centrifugation (10 and 30%) at 1000 rpm at 4°C for 20 min. Thereafter, the cells in 10% interphase as well as between 10 and 30% interphase were recovered, re-suspended, and cultured in DMEM containing 10% FBS, 2 mM L-glutamine, 1% penicillin-streptomycin, and 3.6 mg/ml glucose. Following 24 h of incubation, AECII were treated with the above collected iPSC-CM (100%) and/or TGF-β1 (5 ng/ml) for 48 h, whereas the control cells were released in the normal iPS medium.

### Reverse Transcription-Polymerase Chain Reaction (PCR)

Total RNAs from MEFs, iPS cells and ES cells were prepared by using RNApure Rapid Extraction Kit (BioTeke Corporation, Beijing, China) according to the instructions of the manufacturer. Reverse transcription of equal total RNA was performed using oligo (dT) primer and Super M-MLV reverse transcriptase (BioTeke Corporation) to generate complementary DNA (cDNA). Then, cDNA was amplified by using reverse transcription- polymerase chain reaction (RT-PCR) with the specific primers and Taq PCR Master-mix (BioTeke Corporation), which was performed in Life Express PCR (BIOER, Hangzhou, China). The primer information used is presented in **Table 1**. β-actin was used as the internal control gene. The reverse transcription- PCR conditions were as follows: (1) an initial incubation at 95°C for 5 min, (2) denaturation at 95°C for 20 s, (3) annealing at 54°C for 20 s, and (4) extension at 72°C for 30 s. Steps (2–4) were repeated for 35 cycles, followed by a final polymerization at 25°C for 5 min. Subsequently, the PCR amplification products (Oct4, Sox2, Nanog, Klf4, Fbx15, and β-actin) were visualized in 1.5% agarose gel and analyzed using Gel Imaging Analyzer (Liuyi, Beijing, China).

**Table 1:** Sequences of the primers used for reverse transcription-polymerase chain reaction.

Gene name	Primer sequence (5′–3′)	Product size (bp)
Oct4	F: CCCAACGAGAAGAGTATGAGG	177
	R: GAGCAGTGACGGGAACAGA	
Sox2	F: GCACAGATGCAACCGATGC	167
	R: TCGGACTTGACCACAGAGCC	
Nanog	F: CAGGGCTATCTGGTGAACG	202
	R: CGAAGTTATGGAGCGGAGC	
Klf4	F: CCTACTTATCTGCCTTGCTGATTGTC	142
	R: CCCCCAGATTGCCCGAGAT	
Fbx15	F: GGGATAAAGAAGATGGATACTGG	161
	R: GATTGTCCAACCTAAGCCAGA	
β-actin	F: CTGTGCCCATCTACGAGGGCTAT	155
	R: TTTGATGTCACGCACGATTTCC	

### BLM-Induced Pulmonary Fibrosis in Mice

Male C57BL/6 mice were randomly divided into three groups, including the control group (also the sham group), BLM-induced group (BLM group), and iPS cell treatment group (BLM+iPS group). All mice were anesthetized by intraperitoneal injection with 10% chloral hydrate (3.5 ml/kg) followed by separating trachea from esophagus via blunt dissection. For the induction of pulmonary fibrosis, mice were then given a single intratracheal instillation of BLM (5 mg/kg body weight in 0.1 ml sterile normal saline; Melonepharma, Dalian, China). Meanwhile, the mice in the sham group received an equal volume of sterile normal saline by the same pattern. After 24 h of BLM instillation, the iPS cell treatment group received tail vein injection with 0.5 ml PBS containing 2 × 10^6^ iPS cells, while other mice were given intravenous injection of equal volume of PBS as control. On day 21 after BLM instillation, mice from each group were anesthetized and bronchoalveolar lavage fluid (BALF) collection was performed by lavaging the lung three times with total 1.5 ml aliquots of normal saline via a tracheal catheter. Following sacrifice, lung tissues were rapidly excised. Some right lung were weighted (wet weight), and then dried until a constant weight (dry weight) was obtained. Some right lungs were fixed in 4% paraformaldehyde, and some left tissues were kept at -80°C for subsequent real time-PCR and Western blot analyses. On day 0 and day 21, body weight of each mouse was recorded, respectively.

### Histologic Examination

The paraformaldehyde-fixed pulmonary specimens were dehydrated in gradient ethanol, embedded in paraffin, and sliced into sections at 5 μm. After dewaxing and hydration, the sections were subjected to hematoxylin and eosin (HE) staining and Masson staining. For HE staining, the sections were stained with hematoxylin (Solarbio, Beijing, China) for 5 min and immersed into 1% hydrochloric ethanol. After washing, the sections were counterstained with eosin for 3 min. For Masson staining, the sections were stained with hematoxylin for 6 min and then ponceau acid fuchsin for 1 min. Following treatment with 1% phosphomolybdic acid for 5 min, the sections were counterstained with aniline blue solution. Subsequently, all slices were dehydrated with gradient ethanol, permeabilized in xylene, mounted with resin, and finally observed under a microscope (DP73; Olympus, Tokyo, Japan).

### Enzyme-Linked Immunosorbent Assay (ELISA)

The levels of interleukin (IL)-1β, IL-6, tumor necrosis factor (TNF)-α and TGF-β1 in BALF were determined using respective commercial Mouse ELISA Kits (Boster, Wuhan, China) according to the manufactures’ instructions. After the reaction, the optical density was measured at 450 nm by using a plate reader (ELX-800, BIOTEK, USA).

The collagen content in the mouse lung tissues was examined by collagen I (Col I) assay and hydroxyproline (Hyp) assay using respective commercial Mouse ELISA Kits (Col I kit from USCN, Wuhan, China; Hyp kit from Nanjing Jiancheng Bioengineering Company, Nanjing, China). Left lungs were homogenized in PBS, and the protein concentrations in the supernatants of homogenate were then determined using BCA Protein Assay Kit (Wanleibio, Shenyang, China). Subsequently, all steps of the Col I and Hyp assay were performed according to the protocols of manufacturers. For Col I assay, the absorbance of each sample at 450 nm was measured by a plate reader and the level of lung Col I was expressed as μg/g of wet lung. For Hyp assay, the samples were determined for absorbance at 550 nm using a UV752 spectrophotometer (Yoke, Shanghai, China), and the Hyp content was expressed as μg/mg of proteins.

The levels of prostaglandin E_2_ (PGE_2_) and nitric oxide (NO), two major inflammatory mediators, were measured in the supernatants of lung homogenate by using commercial ELISA Kit (USCN, Wuhan, China) and Griess reagent Kit (Beyotime, Jiangsu, China), respectively, following the instructions provided by the manufacturers. For PGE_2_ assay, the absorbance at 450 nm was determined using a plate reader and the amounts of PGE2 were expressed as pg/mg of proteins. For NO assay, the absorbance of each well was read at 540 nm wavelength, and the values were expressed as μmol/g of proteins.

### Immunohistochemical Staining

For immunohistochemistry, paraffin-embedded pulmonary sections were deparaffinized with xylene, hydrated in gradient ethanol, and microwaved for 10 min for antigen retrieval. After washing with PBS, the sections were incubated with 3% H_2_O_2_ for 15 min to quench the endogenous peroxidase activity and blocked with goat serum (Solarbio, Beijing, China) for 15 min at room temperature. Subsequently, the sections were incubated with primary antibodies against iNOS (1:200) and xyxlooxygenase-2 (COX-2; 1:200) (Boster, Wuhan, China) in a humidified chamber at 4°C overnight. After rinsing with PBS, the slides were incubated with biotinylated goat anti-rabbit secondary antibody (1:200; Beyotime) for 30 min at 37°C and then avidin-biotin- horseradish peroxidase (HRP; Beyotime) for 30 min at 37°C. Finally, the sections were visualized by adding diaminobenzidine (DAB), counterstained with hematoxylin, and examined under light microscope.

### Immunofluorescence Staining

Paraffin-embedded lung tissue sections were deparaffinized and rehydrated with xylene and gradient ethanol, respectively. After antigen retrieval by microwaving for 10 min, the slides were blocked with goat serum for 30 min at room temperature, followed by incubation with primary antibody E-cadherin (1:100; Boster) in a humidified chamber at 4°C overnight. Then, tissue sections were rinsing with PBS and incubated with Cy3-labeled goat anti-rabbit IgG (1:200; Beyotime) in a humidified chamber for 1 h at room temperature. Thereafter, nuclear staining was performed using DAPI (Biosharp, Hefei, China), and tissue sections were mounted in neutral glycerine and observed under a fluorescence microscopy (BX53; Olympus, Tokyo, Japan).

Following treatment, the AECII cultured on coverslips were fixed for 15 min in 4% paraformaldehyde and permeabilized with 0.1% TritonX-100 at room temperature for 30 min. After blocked with goat serum for 15 min, the slides were incubated with the primary antibody E-cadherin (1:200; Boster) in a humidified chamber at 4°C overnight followed by Cy3-labeled goat anti-rabbit IgG (1:200; Beyotime) for 60 min at room temperature. Then, nuclear staining was performed using DAPI. Finally, the slides were mounted in neutral glycerine and observed under a fluorescence microscopy.

### Quantitative Real Time-PCR

Total RNA was extracted from lung tissues by using RNA simple Total RNA Kit (TIANGEN Biotech, Beijing, China) according to the instructions of manufacturer. Equal RNA was subjected to reverse transcription in a reaction system containing M-MLV reverse transcriptase and random primers. Subsequently, quantitative real time PCR was performed using SYBR green Master Mix (Solarbio) on Exicycler 96 Real-Time Quantitative Thermal Block (BIONEER, Daejeon, South Korea). The primers used for quantitative real time PCR are shown in **Table 2**. β-actin served as an internal control to normalize the expression of mRNA. The conditions of PCR were as follows: an initial incubation at 95°C for 5 min, followed by 40 cycles of denaturation at 95°C for 10 s, annealing at 60°C for 20 s, and extension at 72°C for 30 s, and a final incubation at 4°C for 5 min. The relative gene expression analysis was conducted using the 2^-ΔΔCT^ method.

**Table 2:** Sequences of the primers used for real time-polymerase chain reaction.

Gene name	Primer sequence (5′–3′)	Product size (bp)
iNOS	F: GCAGGGAATCTTGGAGCGAGTTG	139


	R: GTAGGTGAGGGCTTGGCTGAGTG


COX-2	F: GATGACTGCCCAACTCCCA	193


	R: TGAACCCAGGTCCTCGCTTA


TGF-β1	F: GCAACAATTCCTGGCGTTACCT	129


	R: GAAAGCCCTGTATTCCGTCTCC
E-cadherin	F: TCAAAGTGGCGACAGACGG	185
’	R: GTTGGATTCAGAGGCAGGGT
β-actin	F: CTGTGCCCATCTACGAGGGCTAT	155
	R: TTTGATGTCACGCACGATTTCC

### Western Blot Analysis

Total protein was extracted from lung tissues or AECII using Total Protein Extraction Kit (Wanleibio) and protein concentrations were determined using BCA Protein Assay Kit (Wanleibio) according to the respective manufacturers’ protocols. Equal amount of protein (40 μg) was separated by 8%-14% SDS-PAGE and transferred onto polyvinylidene fluoride (PVDF) membranes (Millipore). After blocking with 5% non-fat milk or 1% BSA in Tris Buffered Saline with Tween 20 (TBST) for 1 h at room temperature, the membranes were incubated overnight at 4°C with the following primary antibodies: α-SMA (1:400), iNOS (1:400), COX-2 (1:400), E-cadherin (1:400), Fibronectin (1:400) (BOSTER, Wuhan, China), matrix metalloproteinase (MMP)-2 (1:500), MMP-9 (1:500), p-Smad2 (1:500), Smad2 (1:500), p-Smad3 (1:500), Smad3 (1:500), Vimentin (1:500) (Bioss, Beijing, China), or tissue inhibitor of metalloproteinases (TIMPs)-1 (1:200), TIMP-2 (1:200), TGF-β1 (1:200) (Santa Cruz, TX, USA). Subsequently, the blots were washed four times with TBST, followed by incubation with HRP-conjugated goat anti-rabbit IgGs (1:5000; Wanleibio) or goat anti-mouse IgGs (1:5000; Beyotime) for 45 min at 37°C. After washing six times with TBST for 5 min each, the interest proteins were developed using enhanced chemiluminescence (ECL; Wanleibio) and the densitometric analysis was conducted with Gel-Pro-Analyzer system (Liuyi, Beijing, China). Thereafter, the membranes were stripped and re-probed with β-actin antibody (1:1000, Santa Cruz), which served as an internal control.

### Statistical Analysis

The results were presented as mean ± standard deviation (SD) and analyzed using GraphPad Prism Software Version 5.0 (GraphPad Software, Inc., La Jolla, CA, USA). Statistical differences among different groups were assessed by one-way analysis of variance (ANOVA) followed by Newman–Keul’s test. When *p* < 0.05, the results were considered to indicate statistical significance.

## Results

### The Characterization of iPS Cells and their Effects on BLM-Induced Pulmonary Histological Damage in Mice

**FIGURE 1:**
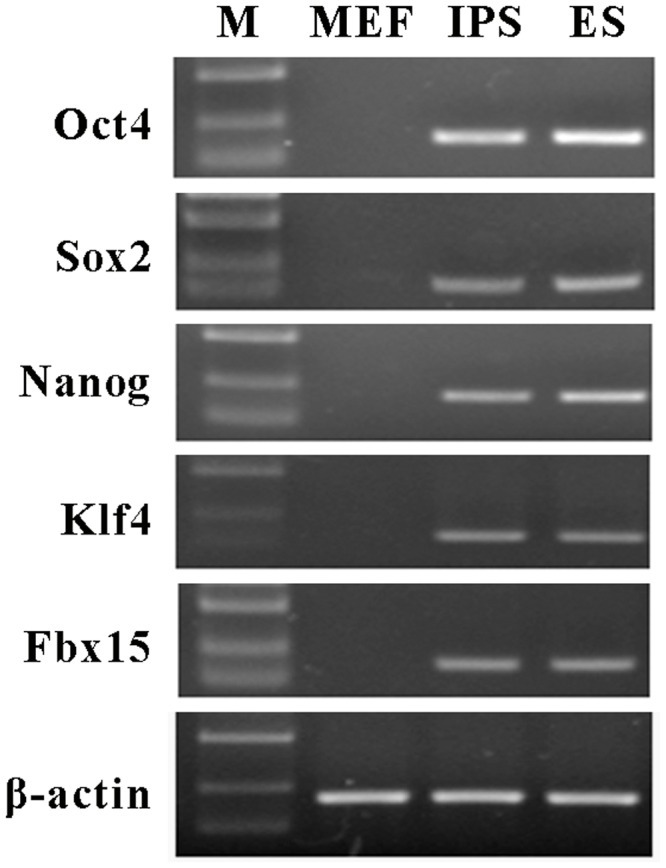
**Characterization of mouse induced pluripotent stem (iPS) cells.** Reverse transcription-PCR was performed to examine the expression of mouse embryonic stem (ES) cells maker genes in iPS cells, including Oct4, Sox2, Nanog, Klf4, and Fbx15. β-actin was used as an internal control. Mouse embryonic fibroblasts (MEFs) were served as the negative control and ES cells were served as the positive control. M represents marker bands.

To generate mouse iPS cells, we performed lentiviral transfection into MEFs with vectors carrying three genes (Oct4, Sox2, and Klf4). After 2 or 3 weeks of culture, iPS cell colonies were detected. The results of reverse transcription-PCR showed that the iPS cells expressed a gene signature similar to ES cell markers, including Oct4, Sox2, Nanog, Klf4, and Fbx15 (**Figure 1**).

**FIGURE 2:**
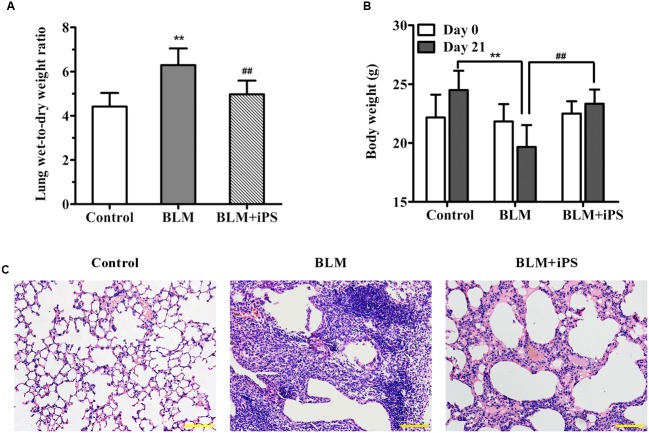
**Effects of iPS cell transplantation on lung histological damage in mice with bleomycin (BLM)-induced pulmonary fibrosis.** All mice except controls were intratracheally instilled with BLM (5 mg/kg), while the controls were received an equal volume of normal saline by the same pattern (also as the sham group). In BLM+ iPS group, mice were i.v. injected with iPS cells after 24 h of BLM instillation. Lung tissues were collected on day 21 after BLM treatment. **(A)** Some right lung lobes were weighted and then dried to obtain a constant weight. The lung wet-to-dry weight ratio was calculated. **(B)** The body weight of each mouse was recorded on day 0 and day 21, respectively. **(C)** Lung tissue sections from control mice, BLM-induced mice and BLM+iPS mice were stained with Hematoxylin and eosin for histological assessment, and the representative images are shown. Original magnification was 200×. Data are expressed as mean ± SD (*n* = 6). ^∗∗^*p* < 0.01 vs. the control group; ^##^*p* < 0.01 vs. the BLM group.

Treatment with BLM led to a significant increment in the lung wet-to-dry weight ratio (W/D) compared with the control group (**Figure 2A**). Nevertheless, the increased W/D of the BLM-induced mice was remarkably reduced by intravenous delivery of iPS cells 24 h after BLM administration. In addition, compared to the control group, BLM administration resulted in an obvious loss of body weight, which was inhibited by the treatment of iPS cells (**Figure 2B**). Histologically, the lung alveoli in the sham group had well-organized hollow cavities with thin lined alveolar septa (**Figure 2C**). On the contrary, markedly histological abnormalities were observed in the lung tissue sections from BLM model group, as shown by obviously collapsed alveolar spaces, thickened alveolar walls, and dense interstitial infiltration by fibroblasts and inflammatory cells. Of interest, the treatment with iPS cells significantly limited inflammatory infiltration and thickened alveolar septa induced by BLM instillation.

### iPS Cells Inhibit BLM-Induced Collagen Deposition in Mice

**FIGURE 3:**
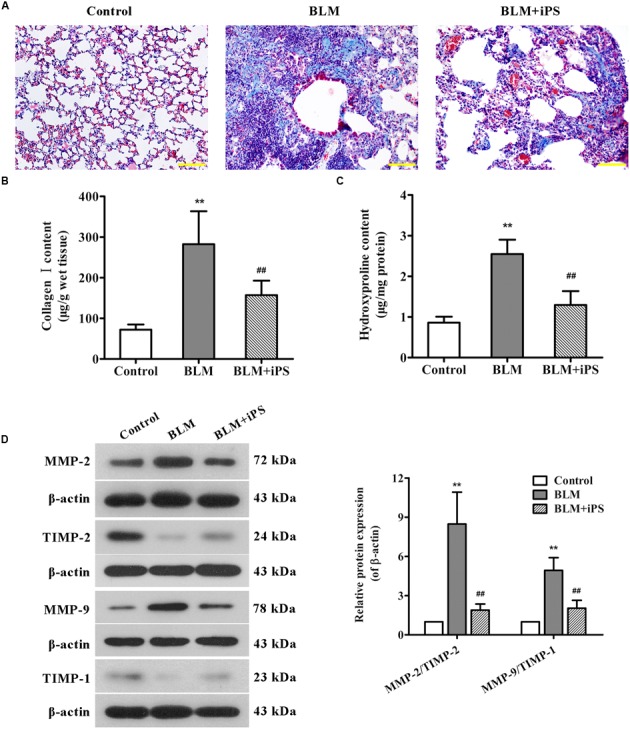
**Effects of iPS cell treatment on BLM-induced collagen deposition in mice. (A)** Pulmonary tissue sections from each group were subjected to Masson’s staining for the visualization of collagen deposition. The representative images are shown. Original magnification was 200×. **(B,C)** Collagen I expression **(B)** and hydroxyproline content **(C)** in mouse lung tissues from each group were determined using ELISA kits. **(D)** The protein expression levels of MMP-2, MMP-9, TIMP-1, and TIMP-2 in lung tissues from each group were determined using Western blot analysis. The representative bands are shown (*left*), and the relative band intensity ratios of MMP-2/TIMP-2 and MMP-9/TIMP-1 were analyzed (*right*). Data are presented as mean ± SD (*n* = 6). ^∗∗^*p* < 0.01 vs. the control group; ^##^*p* < 0.01 vs. the BLM group.

Masson staining was performed to examine collagen fibers in pulmonary tissues, which were presented in blue. As shown in **Figure 3A**, a large quantity of collagen fibers (blue staining), markedly interstitial fibroplasias, and distorted lung morphologies were observed in the lungs from BLM-induced mice compared with the sham mice. In addition, we also assessed the contents of Col I and Hyp in lung tissues of mice, which are considered as fibrotic markers. Compared with the sham group, the lung Col I and Hyp levels were both drastically increased in the BLM model group (**Figures 3B,C**). These data indicated that the pulmonary fibrosis model induced by BLM was successfully established. As expected, iPS cell treatment significantly suppressed the formation of collagen fibers as well as the up-regulation of Col I and Hyp induced by BLM.

It has been described that during the process of fibrosis, MMP-2 and MMP-9 respectively called gelatinase A and gelatinase B are usually up-regulated, which can disrupt basement membranes through degrading Collagen type IV and induce lung tissue remodeling as well as alveolar thickening, resulting in lung fibrosis (Dancer et al., 2011; Van Doren, 2015). Here, the expression levels of MMP-2, MMP-9 and their corresponding tissue inhibitors (TIMP-2 and TIMP-1) were evaluated by Western blot analysis. As indicated in **Figure 3D**, the expression of MMP-2 and MMP-9 was significantly up-regulated in BLM-treated mice compared with the control mice, whereas the expression of TIMP-2 and TIMP-1 was obviously down-regulated. As expected, treating with iPS cells remarkably inhibited BLM-mediated imbalance in the expression ratios of MMP-2/TIMP-2 and MMP-9/TIMP-1.

### iPS Cells Suppress the Lung Inflammatory Response in BLM-Induced Mice

**FIGURE 4:**
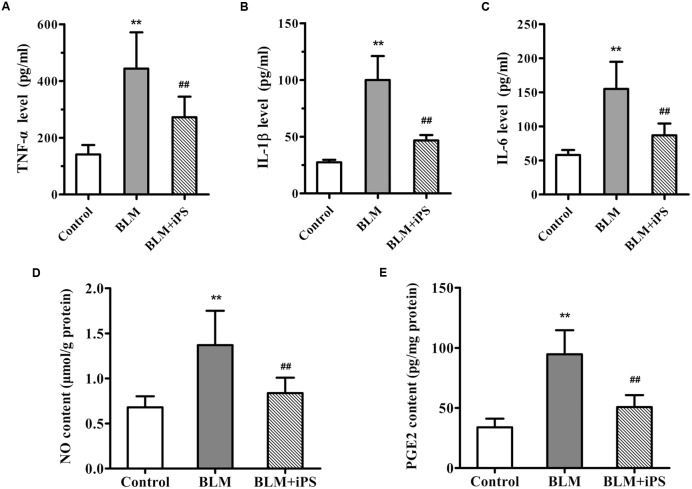
**Effects of iPS cells on the levels of inflammatory cytokines and mediators in BLM-induced pulmonary fibrosis mice. (A–C)** The levels of TNF-α **(A)**, IL-1β **(B)** and IL-6 **(C)** in BALF of each mouse from control group, BLM-induced group, and BLM+iPS group were detected by ELISA kits. **(D,E**) The NO level **(D)** and PGE_2_ content **(E)** in mouse lung tissues from each group were also determined using ELISA kits. Results are expressed as mean ± SD (*n* = 6). ^∗∗^*p* < 0.01 vs. the control group; ^##^*p* < 0.01 vs. the BLM group.

It is well-known that inflammation plays a critical role in the pathogenesis of pulmonary fibrosis. We also observed decreased inflammatory cell infiltration in BLM-induced lung tissues as a result of treatment with iPS cells, the potential mechanisms involved in such anti-inflammatory effect were therefore investigated. As shown in **Figures 4A–C**, a significant increase in the levels of TNF-α, IL-1β and IL-6, several pro-inflammatory cytokines, was observed in the BALF of BLM-induced mice compared with the sham control group. Of note, iPS cell treatment obviously repressed BLM-induced up-regulation of these cytokines in the BALF.

**FIGURE 5:**
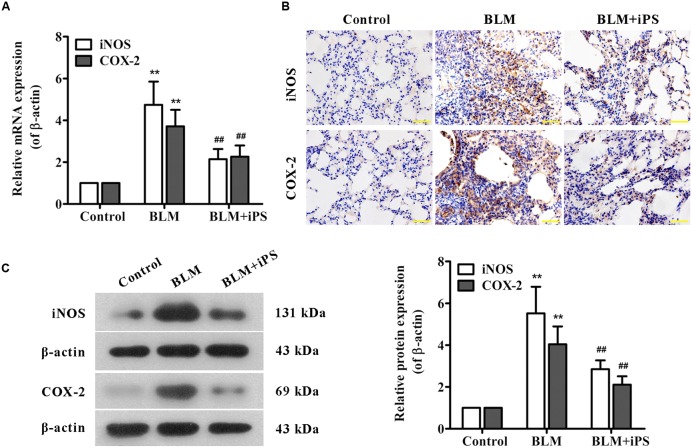
**Effects of iPS cells on the expression of iNOS and COX-2 in lung tissues of BLM-induced mice. (A)** The mRNA expression levels of iNOS and COX-2 in pulmonary tissues from each group were measured using real time PCR. **(B)** The protein expression levels of iNOS and COX-2 in lung tissue specimens from each group were determined by immunohistochemical staining. The representative images are shown. Original magnification was 400×. **(C)** The protein expression levels of iNOS and COX-2 in lung tissues from each group were detected by Western blot analysis. Representative bands are shown (*left*), and the relative band intensity ratio to endogenous control β-actin was quantified (*right*). Data are presented as mean ± SD (*n* = 6). ^∗∗^*p* < 0.01 vs. the control group; ^##^*p* < 0.01 vs. the BLM group.

Considering evidence that overproduction of NO has an essential role for the lung inflammatory response during the development of pulmonary fibrosis (Liu et al., 2012), the level of NO production was detected in the lung tissue samples. As shown in **Figure 4D**, in contrast to the mice in the sham control group, BLM instillation caused a remarkable elevation of NO level in the pulmonary tissues, while such rise was significantly inhibited by treatment with iPS cells. Furthermore, to investigate whether the inhibition of NO production was via suppressing the expression of iNOS, an enzyme that synthesizes NO, iNOS mRNA and protein levels were determined by quantitative real-time PCR as well as Western blot and immunohistochemical analyses, respectively. Consistent with its repression of NO production, iPS cell treatment significantly inhibited BLM-induced up-regulation of iNOS expression at both mRNA and protein levels (**Figures 5A–C**).

PGE_2_, an important inflammatory mediator, is produced from arachidonic acid mainly by COX-2. Several studies have reported that COX-2-induced PGE_2_ has also been linked to the pathogenesis of pulmonary fibrosis (Stratton and Shiwen, 2010; Zhao et al., 2014). The ELISA results indicated that the PGE_2_ level of lung tissues significantly increased in BLM-induced mice compared with the mice in the sham group, but markedly decreased by the following iPS cells treatment (**Figure 4E**). Additionally, data from real time PCR, immunohistochemistry and Western blot analyses revealed that the expression level of COX-2 paralleled that of PGE_2_ in the lung tissues of mice (**Figures 5A–C**). Taken together, these findings indicated that iPS cell treatment could protect the lung against BLM-induced deleterious inflammation.

### iPS Cells Inhibit TGF-β/Smad2/3 Signaling Activated by BLM in Mice

**FIGURE 6:**
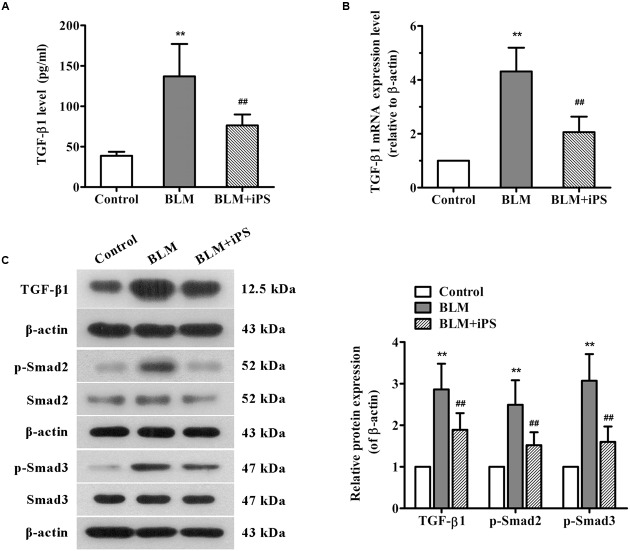
**Effects of iPS cells on TGF-β1/Smad2/3 signaling pathway in the lung tissues of BLM-mediated mice. (A)** The TGF-β1 levels in BALF of each mouse from control group, BLM-induced group, and BLM+iPS group were examined by ELISA kit. **(B)** The mRNA expression levels of TGF-β1 in pulmonary tissues from each group were detected using real time PCR. **(C)** The protein expression levels of TGF-β1, Smad2/3, and p-Smad2/3 in lung tissues from each group were analyzed by Western blot. The representative bands are shown (*left*), and the density values of blots were normalized to the internal control β-actin (*right*). Data are expressed as mean ± SD (*n* = 6). ^∗∗^*p* < 0.01 vs. the control group; ^##^*p* < 0.01 vs. the BLM group.

TGF-β1, a potent profibrotic factor, plays a pivotal role throughout the whole process of pulmonary fibrosis, including lung inflammatory response, fibroblasts proliferation, transformation of fibroblasts to myofibroblasts, and EMT. This study investigated whether TGF-β signaling was involved in the antifibrotic effects of iPS cells. The ELISA results showed that BLM induced a significant increase in TGF-β1 level of BALF which was obviously inhibited after iPS cell treatment (**Figure 6A**). The results of real time PCR and Western bolt analyses also revealed a suppressive effect of iPS cells on TGF-β1 expression in BLM-induced lung fibrosis model (**Figures 6B,C**). Furthermore, we also detected the expression of downstream signaling of TGF-β1, p-Smad2 and p-Smad3 proteins, in the mouse lung tissues. As shown in **Figure 6C**, an increased expression of p-Smad2/3 was observed in BLM-mediated group when compared to the sham control group. Treatment with iPS cells to BLM-induced group led to a significant decrease in the expression of these two proteins. Taken together, these findings suggest that iPS cells could inhibit BLM-activated TGF-β1/Smad2/3 signaling pathway in pulmonary fibrosis mice.

### iPS Cells Repress BLM-Induced EMT in Mice

**FIGURE 7:**
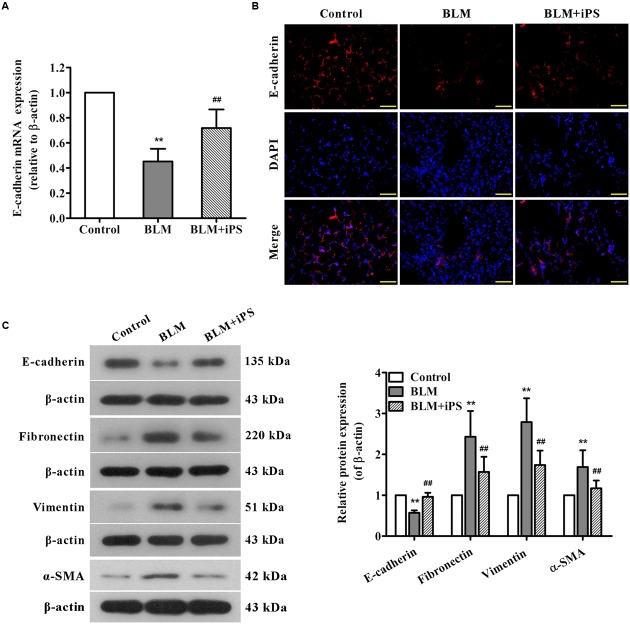
**Effects of iPS cells on BLM-mediated EMT in mice with pulmonary fibrosis. (A)** The mRNA expression levels of E-cadherin in lung tissues from each group were determined using real time PCR. **(B)** Immunofluorescence staining was performed to detect E-cadherin protein expression (red) in lung tissue sections from each group. Nuclei were visualized by DAPI staining (blue). The representative images of each group are presented. Original magnification was 400×. **(C)** The expression levels of EMT-associated proteins in lung tissues from each group were determined using Western blot analysis. The representative bands are shown (*left*), and the relative band intensity ratio to β-actin was analyzed (*right*). Data are expressed as mean ± SD (*n* = 6). ^∗∗^*p* < 0.01 vs. the control group; ^##^*p* < 0.01 vs. the BLM group.

Epithelial to mesenchymal transition, characterized by the conversion of epithelial cells into mesenchymal cells, is well-accepted to be a pivotal process during lung fibrosis. In this work, to further explore the potential role of iPS cells in pulmonary fibrosis, we also investigated their effects on BLM-mediated EMT. The results of real time PCR showed that BLM treatment resulted in a remarkable decrease in the mRNA expression of epithelial marker E-cadherin, while such reduction was markedly repressed by iPS cell treatment (**Figure 7A**). As presented in **Figure 7B**, lung tissues from BLM-induced mice showed less positive cells of E-cadherin staining compared with those from the sham mice. Of note, this decrease of E-cadherin expression was partially suppressed by iPS cell treatment. Western blot analysis confirmed these results observed in immunofluorescence staining (**Figure 7C**). By contrast, the expression levels of mesenchymal markers including fibronectin, vimentin and α-SMA were strongly up-regulated in BLM-induced mice and almost down-regulated to the normal levels in iPS cell-treated mice (**Figure 7C**). Accordingly, these results suggest that iPS cells have inhibitory effect on EMT in BLM-induced pulmonary fibrosis.

### iPSC-CM Suppress TGF-β1-Induced Smad2/3 Signaling and EMT in AECII

**FIGURE 8:**
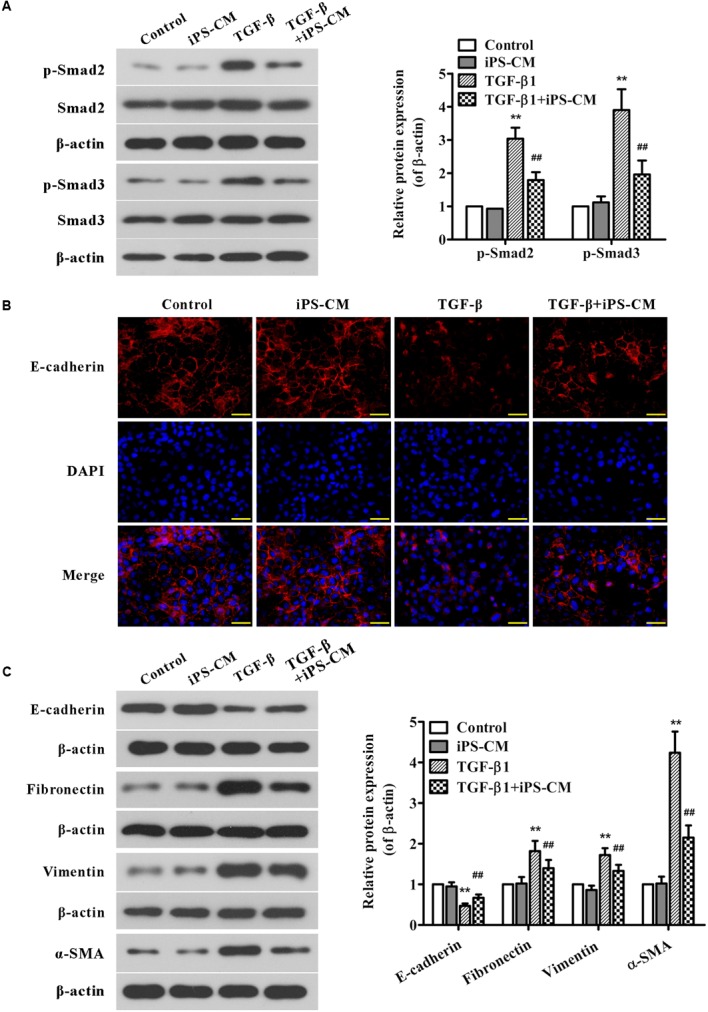
**Effects of iPSC-CM on Smad2/3 signaling and EMT in TGF-β1-induced AECII.** The AECII were treated with the collected iPSC-CM (100%) and/or TGF-β1 (5 ng/ml) for 48 h, whereas the control cells were cultured in the normal iPS medium. **(A)** The protein expression levels of Smad2/3, and p-Smad2/3 in lung tissues from each group were analyzed by Western blot. The representative bands are shown (*left*), and the density values of blots were normalized to the internal control β-actin (*right*). **(B)** Immunofluorescence staining was performed to determine E-cadherin protein expression (red) in AECII from each group. Nuclei were visualized by DAPI staining (blue). The representative images of each group are presented. Original magnification was 400×. **(C)** The expression levels of EMT-associated proteins in AECII from each group were determined using Western blot analysis. The representative bands are shown (*left*), and the relative band intensity ratio to β-actin was analyzed (*right*). Data are expressed as mean ± SD (*n* = 3). ^∗∗^*p* < 0.01 vs. the control group; ^##^*p* < 0.01 vs. the TGF-β1-treated group.

In order to understand the detail mechanism of iPS cells on TGF-β1-Smad signaling and EMT, we treated AECII with iPSC-CM and/or TGF-β1 and then examined the protein expression of smad 2/3 and EMT-associated markers. As shown in **Figure 8A**, treatment with iPSC-CM significantly inhibited the up-regulation of p-Smad2 and p-Smad3 proteins induced by TGF-β1 in AECII. Moreover, the results of immunofluorescence staining showed that AECII treated by TGF-β1 exhibited less positive cells of E-cadherin staining compared with the control cells, which was confirmed by Western blot analysis (**Figures 8B,C**). Additionally, treatment with TGF-β1 strongly up-regulated the expression levels of mesenchymal markers including fibronectin, vimentin and α-SMA in AECII, suggesting induction of EMT by TGF-β1 (**Figure 8C**). Nevertheless, it demonstrated that iPSC-CM treatment remarkably suppressed TGF-β1-induced EMT in AECII (**Figures 8B,C**).

## Discussion

Pulmonary fibrosis is a progressive fibrotic lung disease with high morbidity and mortality for which there is no effective treatment capable of improving or at least suppressing the progressive course. Accumulating evidence highlights the use of stem cell-based therapy for this fibrotic lung disease (Ortiz et al., 2003; Rojas et al., 2005; Ni et al., 2015). In this study, pulmonary fibrosis was induced in mice by intratracheal instillation of BLM, and the potential mechanisms of iPS cells in BLM-induced lung fibrosis were investigated. The present data indicated that administration of iPS cells inhibited lung tissue injury caused by BLM, as evidenced by not only a remarkable reduction in lung W/D weight ratio and collagen deposition but also an obvious inhibition in body weight loss. The results further showed that treatment with iPS cells obviously repressed BLM-induced the release of inflammatory mediators, including TNF-α, IL-1β, IL-6, NO and PGE_2_. The molecular evidence revealed for the first time that TGF-β1/Smad2/3 signaling pathway and EMT were suppressed by iPS cell treatment during BLM-mediated pulmonary fibrosis.

Bleomycin -induced pulmonary fibrosis is the classical available experiment model for human lung fibrosis and widely used for investigating the mechanisms of lung injury and fibrosis (Chen et al., 2013). Growing evidence indicates that excessive cytokine-mediated inflammation plays a key role in the initiation of pulmonary fibrosis in the clinical and animal models (Vasakova et al., 2009; Liu et al., 2012). It has been demonstrated that BLM induces inflammatory cell infiltration and subsequently stimulates these cells to secrete pro-inflammatory cytokines, such as TNF-α, IL-1β, and IL-6 (Scheule et al., 1992), which was consistent with our data. By contrast, treatment with iPS cells significantly suppressed such infiltration of inflammatory cells and release of cytokines, which was in agreement with an earlier report showing down-regulated effects of iPS cells on the levels of pro-inflammatory cytokines in BLM-injured lung tissues (How et al., 2013). Moreover, a number of experimental studies support the notion that overproduction of NO, resulting from iNOS over-expression, contributes to the pathogenesis of BLM-induced pulmonary fibrosis (Galuppo et al., 2010; Di Paola et al., 2011; Galuppo et al., 2011). The findings of the current study also demonstrated that BLM led to a marked increase in NO production and iNOS expression, nevertheless, such increase was significantly repressed upon iPS cell treatment. Therefore, these results suggest that the treatment of iPS cells can inhibit BLM-induced inflammatory responses.

In addition, PGE_2_, produced from arachidonic acid mainly by COX-2, has been suggested to be involved in various inflammatory disorders, and its potential role in lung fibrosis has been increasingly recognized, however, remains controversial. Several studies have reported that exogenously supplying PEG_2_ protected mice against BLM-induced pulmonary fibrosis (Wilborn et al., 1995; Failla et al., 2009; Dackor et al., 2011), whereas some other reports showed the detrimental effects of PEG_2_ in BLM-induced fibrotic process (McCann et al., 2011; Zhao et al., 2014; He et al., 2015). Consistent with the latter view, we found that PEG_2_ level and COX-2 expression were up-regulated after BLM induction. These alterations in lung tissues were significantly suppressed by treatment with iPS cells. Our study differed from other studies indicating the benefits of PEG_2_ in alleviating pulmonary fibrosis (Failla et al., 2009; Dackor et al., 2011). We did not directly administrated PEG_2_ but iPS cells to BLM-treated mice, which thus may be through affecting upstream pathways of PEG_2_ not just itself. Additionally, we measured PEG_2_ levels and COX-2 expression in lung tissues on the day 21, the late phase of lung fibrosis, while other studies either did not assess PEG_2_ levels or only at early phase such as 7 days after BLM induction. Overall, the results of our present study provide evidence that the anti-inflammatory effects of iPS cells on BLM-induced lung fibrosis may be associated with the down-regulation of COX-2-induced PEG_2_. However, the precise mechanisms underlying the regulatory role of iPS cells in the production of PEG_2_ induced by BLM need to be further elucidated.

Recently, TGF-β1 has attracted considerable attention as a potential therapeutic target for lung fibrosis because of its cardinal role in initiation and progression of this fibrosis. It has a potential to activate various pro-inflammatory factors and mediate inflammatory response (Lei et al., 2011). It also caused the deposition of type I and type III collagens in BLM-induced lung fibrosis (Coker et al., 1997). Several studies have demonstrated that the contribution of TGF-β1 to fibrosis is mediated mainly through the Smad-dependent signaling pathway, in which Smad2 and Smad3 are phosphorylated and then translocated into the nucleus where they induce the transcription of target genes (Derynck and Zhang, 2003; Willis and Borok, 2007). Corresponding to previous reports (Zhao et al., 2010; Liu et al., 2012), we observed that BLM induced significant increases in TGF-β1 level of BALF and its expression in lung tissues, whereas transplantation of iPS cells obviously inhibited BLM-activated TGF-β1. In addition, iPS cell treatment suppressed BLM-enhanced phosphorylation of Smad2 and Smad3 in lung tissues. Also, treatment with iPSC-CM significantly inhibited the up-regulation of p-Smad2 and p-Smad3 in TGF-β1-induced AECII. These observations suggest that the anti-fibrotic effects of iPS cells on BLM-induced pulmonary fibrosis may be partly due to the suppression of TGF-β1/Smad2/3 pathway.

During the progression of pulmonary fibrosis, fibroblasts and myofibroblasts are considered as the protagonists, which are mainly derived from alveolar epithelial cells by undergoing EMT (Chapman, 2011). Our data showed that iPS cell administration obviously up-regulated E-cadherin expression and down-regulated the expression of fibronectin, vimentin and α-SMA in lung tissues of BLM-stimulated mice, suggesting that iPS cells might suppress pulmonary fibrosis via inhibiting EMT. Moreover, TGF-β1 plays a predominant role in EMT process and has been recognized as a primary inducer of EMT during pulmonary fibrosis (Chen et al., 2014). Previous studies demonstrated that TGF-β1 could enhance the expression level of zinc-finger transcriptional factor Snail, which subsequently suppressed the transcription of E-cadherin (Thiery et al., 2009; Li et al., 2013). Hence, we speculated that the inhibitory effect of iPS cells on EMT in BLM-induced mice might be associated with the suppression of TGF-β1-Smad signaling activation. To test this speculation, we used Western blot and immunofluorescence analyses to detect the effect of iPSC-CM on EMT in TGF-β1-induced AECII, which is commonly used as a model of pulmonary fibrosis *in vitro* (Alipio et al., 2011). Consistent with the previous studies, we here demonstrated that AECII treated with TGF-β1 showed obvious EMT, as evidenced by the decrease of E-cadherin expression and the increase of fibronectin, vimentin and α-SMA. iPSC-CM treatment significantly repressed TGF-β1-induced EMT in AECII, supporting our speculation. Taken together, our findings suggest that the inhibitory effects of iPS cells on pulmonary fibrosis may partly attribute to the suppression of TGF-β1-Smad2/3-EMT pathway. However, the further research is needed.

## Conclusion

**FIGURE 9:**
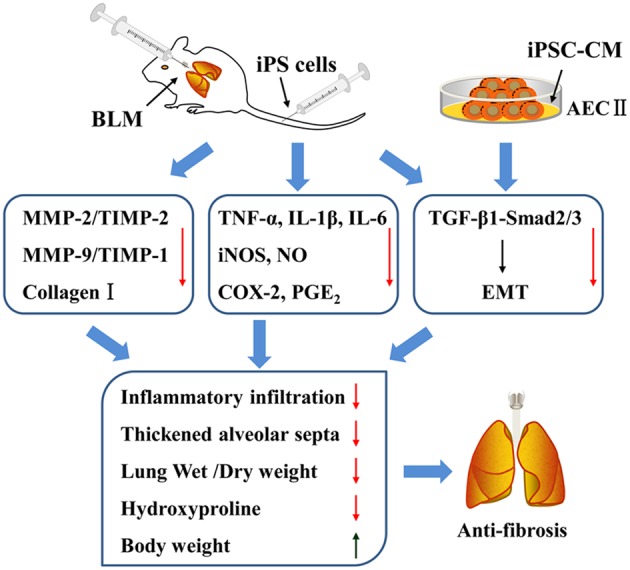
**The summary of effects of iPS cells on pulmonary fibrosis *in vivo* and *in vitro* as well as the involving mechanisms**.

In summary, the present study identified the therapeutic effects of iPS cells in BLM-induced pulmonary fibrosis mice. The potential mechanisms involved in such effects of iPS cells were summarized in **Figure 9**, which were partially mediated by the reduction of inflammatory mediators and inhibition of inflammatory responses in injured lungs. More importantly, our study provided the first line of evidence supporting the notion that transplantation of iPS cells could suppress TGF-β1/Smad2/3 signaling pathway and EMT during BLM-induced lung fibrosis. These results suggest that transplantation of iPS cells is a promising therapeutic strategy for pulmonary fibrosis and provide new insight into its possible mechanisms.

## Author Contributions

YZ and MT conceived and designed the experiments. YZ, ZH, YG, and LZ carried out the animal experiments. YZ, RZ, and XZ conducted the cell experiments. YZ, ZH, and RZ reviewed the data. YZ and MT drafted the manuscript and revised it critically for important intellectual content.

## Conflict of Interest Statement

The authors declare that the research was conducted in the absence of any commercial or financial relationships that could be construed as a potential conflict of interest.
